# Assessment of the Quality of Life in Parents of Children With ADHD: Validation of the Multicultural Quality of Life Index in Norwegian Pediatric Mental Health Settings

**DOI:** 10.3389/fpsyg.2021.638006

**Published:** 2021-02-05

**Authors:** Ingunn Mundal, Petter Laake, Juan Mezzich, Stål K. Bjørkly, Mariela Loreto Lara-Cabrera

**Affiliations:** ^1^Department of Health and Social Sciences, Molde University College, Molde, Norway; ^2^Department of Psychiatry, Kristiansund Community Mental Health Centre, Møre og Romsdal Hospital Trust, Kristiansund, Norway; ^3^Department of Mental Health, Faculty of Medicine and Health Sciences, Norwegian University of Science and Technology (NTNU), Trondheim, Norway; ^4^Department of Biostatistics, Oslo Centre for Statistics and Epidemiology, University of Oslo, Oslo, Norway; ^5^Icahn School of Medicine at Mount Sinai, New York, NY, United States; ^6^San Fernando School of Medicine, San Marcos National University, Lima, Peru; ^7^International College of Person-Centered Medicine, New York, NY, United States; ^8^Centre for Forensic Research, Oslo University Hospital, Oslo, Norway; ^9^Division of Psychiatry, Tiller Community Mental Health Centre, St. Olav’s University Hospital, Trondheim, Norway; ^10^Division of Mental Health, Department of Research and Development, St Olav’s University Hospital, Trondheim, Norway

**Keywords:** Multicultural Quality of Life Index, quality of life, Attention Deficit Hyperactivity Disorder, psychometric properties, well-being, parental QoL, exploratory factor analyses, structural equation model

## Abstract

**Background:** The brief generic Multicultural Quality of Life Index (MQLI) is a culturally informed self-report 10-item questionnaire used to measure health-related quality of life (QoL). QoL is an important outcome measure in guiding healthcare and is held as a substantial parameter to evaluate the effectiveness of healthcare. Attention Deficit Hyperactivity Disorder (ADHD) in children might negatively influence the parents’ QoL. Having a validated questionnaire to measure QoL for this population will therefore be a vital first step in guiding healthcare for parents of children with ADHD. We aimed to examine the reliability and validity of the Norwegian version of the MQLI in a sample of parents of children with ADHD.

**Methods:** In a cross-sectional study, 128 parents of children with ADHD were recruited from four outpatient clinics within the Child and Adolescents Mental Health Services (CAMHS) in Norway. They completed the MQLI along with an alternative well-being scale, the Five-item World Health Organization Well-being Index (WHO-5), and a form including demographic variables. Reliability and validity of the MQLI were examined. We conducted a factor analysis and calculated internal consistency and the correlation between the MQLI and the WHO-5.

**Results:** Factor analysis of the parents reported MQLI yielded a one-factor solution. For the MQLI, Cronbach’s alpha was 0.73. The correlation between the two measures of MQLI and WHO-5 was high (*r* = 0.84), reflecting convergent validity since the association between the two measures was strong.

**Conclusion:** Results from this study support the reliability and validity of the Norwegian version of the MQLI for assessment of quality of life in parents of children with ADHD with good psychometric properties. Study findings support the use of the questionnaire in CAMHS.

## Introduction

Attention Deficit Hyperactivity Disorder (ADHD) typically emerges in childhood with an average prevalence of 5% (Sayal et al., 2018) and accounts for a large proportion of burden of disease in youth (Gore et al., 2011; Dey et al., 2019). With the demands of the treatment and diagnosis, the need for care and support relies on their families in many ways. Parents fill an important role in caring for their child with ADHD and in providing tasks that parents of children without such conditions are not confronted with, such as initiating and supporting professional help seeking (Sayal, 2006), coping with the complexity of ADHD-treatment, and with ADHD having a profound impact on their children’s learning at school (Ghosh et al., 2016; Bonati et al., 2019). Family support is strongly linked to improved health and better psychosocial outcomes for chronically ill children, and the relationship and functioning within the family may change over time coincident with different developmental stages and levels of autonomy (Finzi-Dottan et al., 2011). However, a few recent studies in clinical practice have also documented that ADHD in children negatively affects the parents’ quality of life (QoL), as well as their psychological well-being (Xiang et al., 2009; Andrade et al., 2016; Cappe et al., 2017; Dey et al., 2019). Likewise, parents’ perceived psychological well-being and stress may affect the child’s QoL (Galloway et al., 2019), and the interventions that target parent stress and QoL have the potential of improvements in the child’s QoL as well as enhance their parents’ QoL. Research on ADHD often focuses on child, adolescent, and adult development, leaving parental QoL mainly unexplored (Leeman et al., 2016), and the impact of a care receiver’s disorder on a caregiver has often been captured *via* concepts such as caregiver burden and parenting stress (Zabala et al., 2009; Theule et al., 2010; Dey et al., 2019). Although QoL of parents of children with ADHD is increasingly gaining more attention, and several studies have compared QoL of parents of children with ADHD to QoL of parents of typically general population norms (Xiang et al., 2009; Jafari et al., 2011; Hadi et al., 2013; Zare et al., 2017; Dey et al., 2019), there is a lack of validated tools to measure QoL in this population.

Quality of life is an important outcome measure in guiding healthcare (Mezzich et al., 2011) and is held as a substantial parameter to evaluate the effectiveness of healthcare (Mezzich, 2005). The QoL concept has been defined in many ways. Highlighting the optimal state as one of general well-being in which an individual’s day-to-day functioning across a wide range of domains is influenced by the potentially adverse impact of disease or disorder (Danckaerts et al., 2010). Although a large number of different measures have been designed to capture QoL, there are few short self-reported questionnaires that cover functioning, social, and environmental contexts (Wills, 2007; Linton et al., 2016; Bonnin et al., 2018), and evidence is limited for scales assessing QoL among parents of children with ADHD.

The brief generic Multicultural Quality of Life Index (MQLI) was developed to measure health-related quality of life in different cultures and is based on a critical review of global literature, comprising 10 dimensions of subjective quality of life, including aspects ranging from physical well-being to spiritual fulfillment, and a global perception of QoL (Mezzich et al., 2000). MQLI is a culturally informed instrument and is currently translated into seven language versions, English, Spanish, German, Portuguese, Chinese, Korean, and Greek, and has been validated in different populations and methods of factor analyses (Saletu et al., 2003; Zubaran et al., 2004; Schwartz et al., 2006; Jatuff et al., 2007; Liu et al., 2008; Yoon et al., 2008; Mezzich et al., 2011; Kokaliari and Roy, 2020). It was developed in response to the assessment issues and the need for a multidimensional and comprehensive framework as well as wide applicability, self-assessment, ease of use, and sound psychometric features, which are key characteristics that instruments designed to assess quality of life should have (Mezzich et al., 2011).

Both the Spanish and the English version of MQLI have shown high-discriminant validity and differentiates well between samples with different levels of expected quality of life in patients vs. professionals with a high test-retest reliability (*r* = 0.87), also documenting a high internal consistency (Cronbach’s alpha of 0.92) and a factor analysis with a strong factor structure (Schwartz et al., 2006; Mezzich et al., 2011). However, the psychometric properties of the MQLI have been rarely examined among parents in mental health settings and warrant further research.

The measurements of well-being, broadly defined as “the quality and state of a person’s life,” often differ by discipline and are frequently confused with related topics such as health-related quality of life as well as happiness and wellness (Linton et al., 2016). Both the concept of QoL and well-being concern evaluative judgments, meaning that each is an evaluation of life. However, the concepts are not necessarily definite entities, even though they attempt to be concrete (Gasper, 2010). The Five-item World Health Organization Well-being Index (WHO-5) is, for example, a validated outcome measure tool designed to assess self-reported patient well-being and is among the most widely used brief questionnaires assessing subjective psychological well-being as well as quality of life across a wide range of study fields (Bech et al., 2003; Topp et al., 2015), also documenting a high internal consistency (Cronbach’s alpha of 0.92; Lara-Cabrera et al., 2020).

Although several studies have reported that the MQLI is an appropriate tool for the assessment of QoL in routine practice, more research is required to further explore the appropriateness of the MQLI in different settings. Key factors facilitating the MQLI’s cross-cultural adoption include strong beliefs that MQLI grasps the multidimensional framework, the need for cultural suitability, and emphasizing the role of subjectivity in the assessment (Mezzich et al., 2000). Therefore, it is crucial to have generic instruments which assess the health-related, cross-cultural, and subjective QoL. In addition, to be able to draw the right conclusion, the reliable use of questionnaires in clinical settings to actually measure the proposed phenomena, together with adherence to good methodology throughout the process are of utmost importance to ensure reliable and valid results from studies using patient-reported outcome measures (Willert et al., 2015). In order to enable the right conclusion to be drawn, validation is of importance. There is currently no evidence to support the suitability of the Norwegian version of the MQLI for the specialized psychiatric care. Based on findings from empirical research suggesting that the MQLI measures the construct of quality of life, we also hypothesized that the MQLI shows a strong correlation with the WHO-5. The aims of this study were (1) to analyze and compare factor structure, (2) to estimate internal consistency, and (3) to calculate the association between the short Norwegian translation of self-reported MQLI and WHO-5 questionnaires for use in parents of children with ADHD within CAMHS (convergent validity).

## Materials and Methods

### Study Design

This study is a cross-sectional assessment aiming to examine the convergent validity and internal consistency of the Norwegian version of the MQLI questionnaire and includes two questionnaires. The MQLI was used to evaluate the multicultural quality of life and the WHO-5 was used to assess well-being in parents of children with any type of ADHD within CAMHS. Participation was anonymous and voluntary, and only parents of children diagnosed with ADHD were included in this study.

### Participants and Procedure

Norwegian-speaking parents (*n* = 128) of children with ADHD were recruited from four outpatient clinics within CAMHS. The 10-item MQLI and the WHO-5 questionnaires and a few demographic questions were used in the data collection. Consent implied that parents received oral and written information and agreed in that they filled out and returned the questionnaires. The questionnaires were administered as paper and pencil questionnaires.

Parents of children newly diagnosed with ADHD were included between February and May 2019 in four CAMHS in Mid-Norway in the context of their attendance in a 1-day peer co-led parental educational ADHD specific course, which they had signed up for in advance. The diagnostic processes and routine assessments were accomplished according to the Diagnostic and Statistical Manual of Mental Disorders version IV (DSM-IV) as well as the Norwegian national guidelines for ADHD. The latter includes information from patients, parents and teachers, developmental history, somatic health status, and school functioning (APA, 2013; Helsedirektoratet, 2016; Nøvik et al., 2020). Assessments of emotional and behavioral problems were achieved with the Achenbach system of empirically based assessment (ASEBA) checklists (Achenbach, 2009) and ADHD symptoms by the ADHD Rating Scale-IV (ADHD-RS-IV; DuPaul et al., 2016). Also, IQ scores and adaptive functioning were obtained using Wechsler Intelligence Scales for Children (Wechsler, 2003) and Children’s Global Assessment Scale (CGAS; Shaffer et al., 1983). The project team members showed up before the parental course started and handed out questionnaires as well as they briefly and orally informed the parents about the project. Those who were interested and received an anonymous envelope with the questionnaires, which they were asked to complete within the end of the course. Consent was implicitly given by anonymously responding to the questionnaires and returning the envelope at the end of the course. The study was approved by the Regional Committee for Medicine and Health Research Ethics in Mid-Norway (ref.: 2018/1196).

### Translation

The original scale developers of the 10-item MQLI, Mezzich et al. (2011) provided consent for the cross-cultural adaption. Based on the English version of the MQLI (Mezzich et al., 2011), the English version of the MQLI was translated into the Norwegian language followed the standard forward-step and backward-step procedure. First, the MQLI was translated into Norwegian and then back into English by a professional translator (Guillemin et al., 1993; Wild et al., 2005). Then the Norwegian version was tested in a subgroup of user representatives from mental health patient organizations. The items were adapted following the feedback from the user representatives. The item related to spirituality required a deeper discussion and reflection. After two meetings, we managed to facilitate and conceptualize the questionnaire to be suitable for the Norwegian language.

### Measurements

#### The Multicultural Quality of Life Index

Quality of life was measured with the self-rated 10-item MQLI, which is designed to have wide applicability as that it should be useful and relevant for diverse populations and settings, including people undergoing both general medical and psychiatric conditions. It covers key aspects of 10 life dimensions; physical and emotional well-being, independent functioning, occupational functioning, and interpersonal functioning, social and community support, spiritual fulfillment, and finally global perception of QoL, as well as a brief explanation of each concept presented in parentheses (Mezzich et al., 2000, 2011). Each of these 10 items is rated on a 10-point scale ranging from 1 indicating “poor” to 10 indicating “excellent,” with the final score obtained by computing the average of the scores of all items rated by all the individuals, and summing range 10–100 (Mezzich et al., 2011). The respondents were asked to indicate the quality of their health and life by placing an X of any of the 10 point-scale.

#### The World Health Organization 5-Item Well-Being Index

To assess if the MQLI was associated with psychological wellbeing, the WHO-5 was used. The WHO-5 was originally developed to assess the quality of care and subjective well-being among medical patients (Dadfar et al., 2018) and is a generic, self-reported scale including five Likert-type statements to evaluate psychological well-being (Topp et al., 2015). This short questionnaire has adequate validity both as a screening tool for depression and as an outcome measure in clinical trials (Topp et al., 2015). It has the potential for assessing patient outcome and monitoring response treatment in psychiatric care (Newnham et al., 2010; Bech et al., 2018).

The respondents were asked to rate their agreement over the previous 2 weeks on each of the items rated on a 6-point scale from “all of the time” to “at no time” transformed onto a scale from 0 to 100 (high scores indicate better well-being). The WHO-5 captures emotional well-being and contains five positively worded items: “I have felt cheerful and in good spirits,” “I have felt calm and relaxed,” “I have felt active and vigorous,” “I woke up feeling fresh and rested,” and “My daily life has been filled with things that interest me.” The WHO-5 provides brief measures of global well-being and is not time-consuming (Linton et al., 2016).

### Statistical Analysis

To explore the group convergent validity of the MQLI, we performed an exploratory factor analysis (EFA) to assess the numbers of factors and to support dimensionality and interpretation of the factors. Exploratory factor analysis examines how many latent factors which soundly may be considered to summarize the information found in the items. A structural equation model (SEM) was estimated to confirm any association between MQLI and WHO-5.

Kaiser-Meyer-Olkin (KMO) measure of sampling adequacy was found appropriate for conducting EFA if KMO was above 0.5 and Bartlett’s test of sphericity showed a significant *p* < 0.001, confirming correlation among the included variables and that the factor analysis is appropriate (Williams et al., 2010). The factors generated by EFA were accepted if the Eigenvalues were >1, according to Kaiser’s criterion. The hypothesis used for the convergent validation was tested with Pearson’s correlation coefficient. The internal consistency reliability of the questionnaires was evaluated using Cronbach’s alpha coefficient, expecting coefficients above 0.70 (Mokkink et al., 2010).

The evaluation parameters included were (a) internal structure analyses including internal consistency analyzed *via* Cronbach’s alpha coefficient for the 10 items of the instrument, (b) factorial structure analyzed using exploratory factor analysis as extraction method, and (c) SEM analyses estimating the relationship between MQLI and WHO-5.

We assessed the degree to which the scores of the MQLI were consistent with several pre-defined hypotheses. Previous studies have reported a one-factor structure for the MQLI and a unidimensional structure of the questionnaire was thus hypothesized. We also expected *a priori* that the MQLI would show a high correlation with the WHO-5, with a Pearson’s correlation coefficient that would be >0.5 as both scales capture health related quality of life.

Descriptive statistics included means, standard deviations, and percentages of the demographic characteristics of the parents. All statistical analyses were performed using Stata statistical software version 16.0 (StataCorp, 2019). Statistical significance was considered as *p* < 0.05.

## Results

A total of 128 (60%) parents of children with ADHD, including 77 mothers (61%) and 49 fathers (39%), filled in a questionnaire including MQLI and WHO-5. Correcting for missing data, 95% (*n* = 121) of the parents fully completed the questionnaires. Characteristics of the participants are shown in Table 1. Attrition analysis was unattainable because of the anonymous design. Descriptive scores of the items in MQLI are shown in Table 2. The overall internal consistency reliability (Cronbach’s alpha coefficient) for the 10 items of the MQLI was 0.73 indicating that the scale measures only one concept. For the WHO-5, the overall Cronbach’s alpha was 0.89.

**Table 1:** Sample characteristics of parents.

	Caregivers ADHD child (*n* = 128)
**Age of parent (SD)**	39,6 (7.22)
**Parent (%)**	
Mother	77 (61.1)
Father	49 (38.9)
**Gender of child (%)**	
Girl	43 (34.7)
Boy	81 (65.3)
**Age of child (SD**)	10.4 (3.18)
**Marital status (%)**	
Single/divorced/separated	29 (22.8)
Married/cohabitant	98 (77.2)
**Education in years (%)**	
Primary school	11 (8.6)
High school	52 (40.6)
College/University <4 years	65 (50.8)
**Working status (%)**	
Full time work	94 (73.4)
Student	7 (5.5)
Unemployed	10 (7.8)
Part time or sick leave	17 (13.3)
**First language (%)**	
Norwegian/Scandinavian	126 (98.4)
Other	2 (1.6)
**Living place (%)**	
City	60 (48.8)
Village	63 (51.2)

ADHD, attention deficit hyperactivity disorder and SD, standard deviation.

**Table 2:** Descriptive statistics of MQLI items (*n* = 121).

Item	Mean	Standard deviation	Factor loadings
1. Physical well-being	6.62	2.24	0.856
2. Psychological/emotional well-being	6.53	2.19	0.839
3. Self-care and independent functioning	8.09	1.98	0.746
4. Occupational functioning	7.74	2.34	0.802
5. Interpersonal functioning	7.87	1.75	0.783
6. Social emotional support	7.15	2.02	0.741
7. Community and services support	7.16	2.01	0.697
8. Personal fulfillment	6.91	2.16	0.852
9. Spiritual fulfillment	5.69	2.53	0.540
10. Global perception of quality of life	8.47	10.00	0.291

MQLI, Multicultural Quality of Life Index. Mean scores by items and factor loadings of the Norwegian version of the MQLI in parents of children with ADHD.

### Exploratory Factor Analysis of MQLI

**Figure 1:**
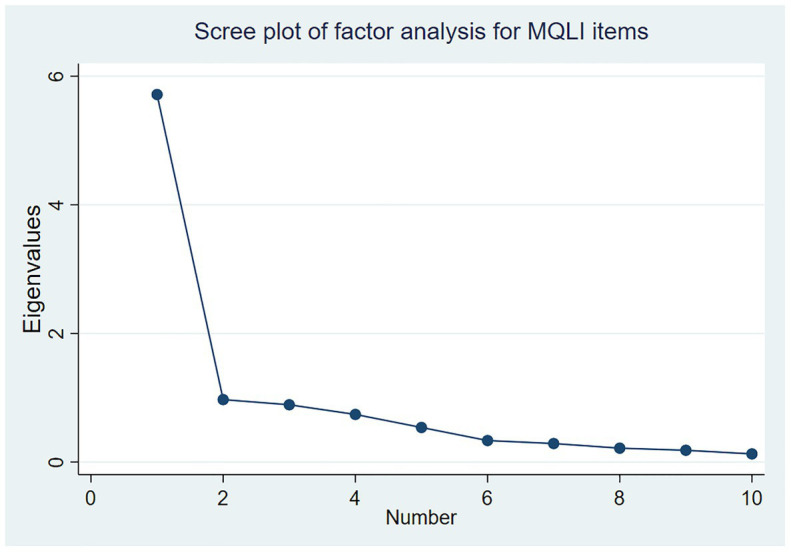
Scree plot of the factor analysis for the MQLI items.

In our study, the eigenvalue criterion suggested an extraction of one factor. The unrotated Exploratory Factor Analysis (EFA) was conducted on the 10 items of the Norwegian translated MQLI and generated one component with Eigenvalues fulfilling Kaiser’s criterion >1 (Kaiser and Michael, 1977), explaining 89.9% of the variance, indicating unidimensionality of the instrument for quality of life and with factor loadings from 0.291 to 0.856. The scree plot of the factor analysis of the MQLI items suggested that the inflection point was a one-factor solution (see Figure 1), which also was the case for the WHO-5 (data not shown). Kaiser-Meyer-Olkin (KMO) measure of sampling adequacy for the items for use in EFA was 0.886 (*p* < 0.001), which indicates a high value above the recommended values of 0.800 and *p* < 0.05. Bartlett’s test of sphericity was significant ( = 814.05, *p* < 0.001) and showed sufficiently large correlations between the items for including them in the EFA (Williams et al., 2010).

### Internal Consistency of MQLI

The overall Cronbach’s alpha of the scale was *α* = 0.73, varying from 0.68 to 0.92, and the item-rest correlation for item representing the Global perception of quality of life was *α* = 0.28, indicating that the association with this item and the others is low. However, the overall inter-item correlation was above 0.5 (*r* = 0.50). Deleting the item Global perception of quality of life improved overall Cronbach’s alpha to 0.92 and the inter-item correlation increased to *r* = 0.57.

### Structural Equation Modeling Analysis of MQLI and WHO-5

**Figure 2:**
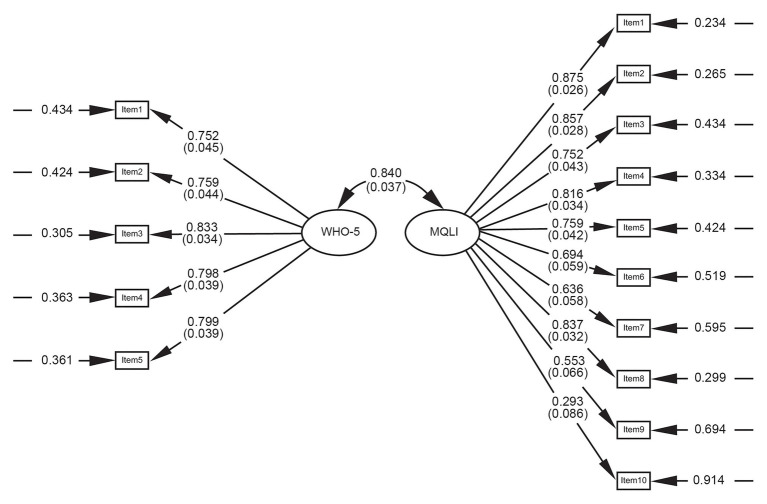
Structural equation model (SEM) for the MQLI and WHO-5. The numbers connected to the arrows from the factors to the items are the loadings (with standard errors). The numbers connected to the arrows pointing to the items are unique variances. MQLI: Multicultural quality of life. WHO-5: The World Health Organization 5-item well-being index.

To assess the relationship and dependencies between the Norwegian version of MQLI and WHO-5, we used a structural equation model (SEM) to examine the degree to which patterns of means and covariation could mirror the conceptual model of quality of life. We found that both the MQLI and WHO-5 reflected the same concept of QoL, and the MQLI scale was positively correlated to the WHO-5 (*r* = 0.84). High correlations between the measurements would indicate high convergent validity. Figure 2 presents the factor structure, the factor loadings, the unique variances for each item of the MQLI and the WHO-5, together with the correlation between the MQLI and the WHO-5.

## Discussion

Quality of life measurements are important among the reported outcome measures of parents of children with ADHD (Linton et al., 2016). In this study, we aimed to examine the reliability and validity of the Norwegian version of the MQLI in a sample of parents of children with ADHD, through analyzing and comparing factor structure, estimating internal consistency, and calculating the association between the MQLI and WHO-5 based on questionnaires for use in parents of children with ADHD within CAMHS.

Our results showed high internal consistency reflecting the concept of QoL. The one-factor structure and the evidence of convergent validity were demonstrated by a strong positive correlation between the MQLI and the WHO-5. Regarding factor structure of the Norwegian MQLI, one component was found with Eigenvalues explaining 89.9% of the variance, together with the scree plot of MQLI suggesting that the inflection point was a one-factor solution. This finding indicated the unidimensionality of the instrument around quality of life. Factor analyses in an earlier study showed a good fit for both the one- and two-factor structures (Álvarez et al., 2010); however, most validation studies provide evidence for one-single factorial solution.

The SEM-analysis between MQLI and WHO-5, examining the degree to which patterns of means and covariation could mirror the conceptual model of quality of life, found that both scales reflected the same concept of QoL. The positive and high correlation (*r* = 0.84) between these concepts supported the convergent validity of the MQLI. As previously found by Álvarez et al. (2010), the MQLI has an adequate convergent validity according to the high correlations observed with the World Health Organization Quality of Life, brief version. These findings, together with acceptable internal consistency and consistent with other translations (Saletu et al., 2003; Zubaran et al., 2004; Schwartz et al., 2006; Jatuff et al., 2007; Liu et al., 2008; Yoon et al., 2008; Kokaliari and Roy, 2020), support the evidence of convergent validity of the MQLI.

One of the main objectives of developing the MQLI was to develop an easy and applicable instrument useful for different ethnic groups and to facilitate a culture-informed and self-rated assessment (Mezzich et al., 2000, 2011; Álvarez et al., 2010). The measurement of a generic and not disease-specific measurement of health-related QoL as an estimate of well-being is of increasing importance, also with a view to the evaluation of parental health and treatment efficacy. A meta-analysis (2010) examining the association between parenting stress and ADHD, found that parents of children with ADHD experienced more parenting stress than parents of nonclinical controls, and that severity of ADHD symptoms, child-occurring conduct problems, and male gender, were associated with parenting stress (Theule et al., 2010). Still, one review found that fathers of children with ADHD experienced less parenting stress (McCleary, 2002). Another recent systematic review of QoL of parents of mentally ill children included seven studies of mainly mothers of children with ADHD, recruited *via* outpatient clinics and compared with parents of healthy children (Dey et al., 2019), showing that parents of older children had lower QoL than parents of younger children. They also showed that parents of mentally ill children are experiencing a compromised QoL relative to parents of healthy children (Dey et al., 2019).

Recent studies have shown that ADHD in children negatively affect their parents’ QoL, especially in terms of psychological well-being, personal fulfillment, couple relationship, and daily life activities, however, perceiving the situation as challenging rather than as a threat or loss, predicted better QoL (Cappe et al., 2017; Sankey et al., 2019). Peer co-led parental educational ADHD specific programs under the auspices of health personnel and user-representatives are training programs that may improve parenting skills and perception concerning their child’s problem, recommending coping strategies that give rise to a better QoL (Xiang et al., 2009; Cappe et al., 2017). However, to improve QoL in parents having a child with ADHD, health personnel should pay more attention to this concern and further investigate parents’ QoL with the MQLI questionnaire to identify the most serious domains of QoL. Thus, the educational ADHD specific programs for parents may be developed and organized to be appropriate for guiding parents to coping strategies, known to be effective in the long term (Lai and Oei, 2014) and grasp the topics that the parents claim as important to them. Assessing the outcomes of interventions in mental health care, such as ADHD specific educational programs for parents, is important and challenging. It is important because producing significant outcomes, i.e., health gains attributable to an intervention, is the main goal of mental health services (Thornicroft and Slade, 2014). In addition, the QoL of parents of children with ADHD should be considered and addressed by health professionals who are in contact with them to develop intervention aiming to build up for the parents QoL and health and keep in mind important associated contextual factors (Da Paz and Wallander, 2017; Dey et al., 2019).

### Strengths and Limitations of the Study

The main strength of this study is that it provides, for the first time, the validation of a QoL measure for parents of children diagnosed with ADHD. The questionnaire achieved robust psychometric properties, so the findings strengthen the current knowledge of the MQLI as a reliable questionnaire for use among this population. Since our study sample comprised parents from four children and adolescent outpatient clinics, this strengthens the generalizability of the results.

An important question concerning the use of the MQLI is the feasibility and applicability of the questionnaire. The MQLI was reported to be easy to administer and was completed within 3 min, however, we do not provide details for item understanding or burden. Our sample size was satisfactory, although the majority of the respondent were mothers, which may challenge the generalizability to parents, as examined in other studies in which fathers and mothers have been shown to differ in some domains in their QoL (Allik et al., 2006; Mugno et al., 2007; Dey et al., 2019).

Although the MQLI is brief and has a high applicability, the study has some limitations that need to be considered. Due to the cross-sectional design of the study, it was not possible to evaluate the test-retest reliability of the measure, and such evidence of reliability testing should be the subject of future studies. Another limitation is that QoL of parents of children with ADHD should be compared with the QoL of parents of healthy children, since they might also experience a slight reduction in their QoL relative to the general population. Future studies could integrate the severity of the individual child’s ADHD condition at the time of QoL measurement of parents are called upon. Comparisons on the QoL of parents of children with ADHD with norm values (i.e., QoL data from the general population), should also be considered, keeping in mind that the effect for this comparison might be slightly overestimated (Dey et al., 2019). A third limitation is the lack of information of parent ADHD. Generally, up to one half of the children with ADHD have one parent with ADHD (Johnston et al., 2012). Parent ADHD is associated with parenting problems, which may be expressed in several ways, depending on the levels of parent symptoms and differential relations in parenting (Williamson et al., 2017). Thus, parent ADHD is reciprocally related to child vulnerabilities, family context and QoL. Moreover, longitudinal studies are required to focus on parents QoL, which should focus on parents’ QoL in families with parent ADHD and child ADHD outcomes by addressing the important aspects of parenting, including gender differences in parents and their children.

### Clinical Relevance

The impact of the child’s ADHD on parents QoL goes beyond the ADHD symptoms. QoL is acknowledged as a key outcome of chronic health conditions and is increasingly used and recommended for clinical care (Puka et al., 2020). The use of MQLI may help health personnel focusing on the outmost importance of family environment, the compromised QoL of parents of children with ADHD, and parent’s needs. Assessing the aspects of QoL in clinical practice is recommended and considered as an important aspect of thorough care (Puka et al., 2020). The application of this questionnaire in everyday clinical practice may include monitoring parents’ quality of life and well-being, which aids a clinical understanding of crucial importance for children with ADHD and their families. The MQLI may also be included to evaluate the effect of parental interventions on quality of life, as well as investigating which parents benefit most from these interventions (Mundal et al., 2020).

### Future Perspectives

A majority of mothers participating in ADHD studies is also found in studies of parent ADHD, where most studies of parent ADHD have also focused on mothers (Williamson et al., 2017; Dey et al., 2019). However, studies show contrasting findings of different risk effects regarding parental gender and level of parents ADHD symptoms on child outcomes (Agha et al., 2013; Williamson et al., 2017). It has been argued that the focus on mothers participating in ADHD studies is due to them being more involved in the caregiving process than fathers (Dey et al., 2019). Thus, the QoL of both male and female caregivers should be elaborated in further studies including parents with ADHD.

More studies are needed to measure QoL and compare parents’ groups in clinical samples, as well as to compare with age- and gender-comparable national norms. It also seems important to have more research focusing on fathers to provide a broader understanding of their QoL. Future studies should employ larger samples, as well as systematic methods for assessing both demographic factors and diagnostic outcomes. These studies should compare whether the quality of life differs for parents of children who have an ADHD-diagnosis and those who do not. Critically, the present findings highlight the need to develop and implement interventions to improve the QoL of the parents.

## Conclusions

Our study findings suggest that the Norwegian version of the MQLI has robust psychometric properties. The MQLI has high internal consistency and can be interpreted in terms of a single factor, as well as having an adequate convergent validity with a high correlation with the WHO-5. Thus, the Norwegian version can be recommended for use to measure quality of life in parents of children diagnosed with ADHD. Additionally, we recommend future research to investigate the psychometric properties of MQLI in other populations and to assess the impact of analyzing studies measuring parental QoL and to highlight parental, child, and contextual QoL associated factors.

## Data Availability Statement

The datasets used and/or analyzed during the current study are available from the first author on reasonable request.

## Ethics Statement

The study was carried out in accordance with the code of ethics of the Declaration of Helsinki, and all procedures and consent forms were reviewed and approved by the Regional Committee for Medicine and Health Research Ethics in Mid-Norway (ref.: 2018/1196). Consent implied that parents received oral and written information and agreed in that they anonymously filled out and returned the questionnaires. Information including personal details was not collected, and the questionnaires did not include names or any other identifying information. Thus, the confidentiality and anonymity were carefully ensured.

## Author Contributions

IM and MLL-C designed the study, collected the data, and drafted the first manuscript. PL performed the statistical analyses and prepared Figures 1 and 2. JM has developed the questionnaire. SB provided quality assessment and guidance. Each version of the draft was circulated to all authors for comments and endorsement of the consensus, and all authors contributed to drafting, interpretation, and critically revising the paper. All authors have read and approved the manuscript to be published and agreed to be accountable for all aspects of the work.

### Conflict of Interest

The authors declare that the research was conducted in the absence of any commercial or financial relationships that could be construed as a potential conflict of interest.

## Abbreviations

ADHD: Attention deficit with hyperactivity disorder
CAMHS: Child and adolescents mental health services
EFA: Exploratory factor analysis
KMO: Kaiser-Meyer-Olkin
MQLI: Multicultural Quality of Life Index
SD: Standard deviation
SEM: Structural equation model
QoL: Quality of life
WHO-5: Five-item World Health Organization Well-being Index
